# USP7 reduces the level of nuclear DICER, impairing DNA damage response and promoting cancer progression

**DOI:** 10.1002/1878-0261.13543

**Published:** 2023-11-02

**Authors:** Xiaojia Liu, Runhui Lu, Qianqian Yang, Jianfeng He, Caihu Huang, Yingting Cao, Zihan Zhou, Jiayi Huang, Lian Li, Ran Chen, Yanli Wang, Jian Huang, Ruiyu Xie, Xian Zhao, Jianxiu Yu

**Affiliations:** ^1^ Department of Biochemistry and Molecular Cell Biology, Shanghai Key Laboratory of Tumor Microenvironment and Inflammation Shanghai Jiao Tong University School of Medicine China; ^2^ Department of Biomedical Sciences, Faculty of Health Sciences University of Macau China

**Keywords:** cancer progression, DICER, MDM2, ubiquitination, USP7

## Abstract

Endoribonuclease DICER is an RNase III enzyme that mainly processes microRNAs in the cytoplasm but also participates in nuclear functions such as chromatin remodelling, epigenetic modification and DNA damage repair. The expression of nuclear DICER is low in most human cancers, suggesting a tight regulation mechanism that is not well understood. Here, we found that ubiquitin carboxyl‐terminal hydrolase 7 (USP7), a deubiquitinase, bounded to DICER and reduced its nuclear protein level by promoting its ubiquitination and degradation through MDM2, a newly identified E3 ubiquitin‐protein ligase for DICER. This USP7‐MDM2‐DICER axis impaired histone γ‐H2AX signalling and the recruitment of DNA damage response (DDR) factors, possibly by influencing the processing of small DDR noncoding RNAs. We also showed that this negative regulation of DICER by USP7 via MDM2 was relevant to human tumours using cellular and clinical data. Our findings revealed a new way to understand the role of DICER in malignant tumour development and may offer new insights into the diagnosis, treatment and prognosis of cancers.

## Graphical abstract

This study demonstrated that, in the nucleus, USP7 downregulates DICER at the protein level, forming a new regulatory axis that impairs DNA damage response and then promotes tumour growth. Additionally, MDM2 is identified as a new ubiquitin E3 ligase of DICER, indirectly mediating the negative regulation of DICER by USP7.

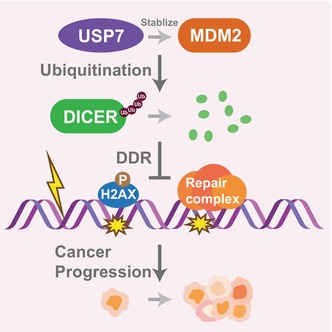

**Abbreviations**
AGO: ARGONAUTE protein
BRCA: breast cancer
CPT: camptothecin
CPTAC: clinical proteomic tumour analysis consortium
DDR: DNA damage response
DDRNA: DNA damage response RNA
DEG: differentially expressed gene
DSB: DNA double‐strand break
dsRNA: double‐stranded RNA
GO: gene ontology
GSEA: Gene Set Enrichment Analysis
GSVA: Gene Set Variation Analysis
HAUSP: herpes‐associated USP
IF: immunofluorescence
KEGG: Kyoto Encyclopedia of Genes and Genomes
miRNA: microRNA
miRNA‐Seq: high‐throughput miRNA sequencing
MITF: microphthalmia‐associated transcription factor
MRN: MRE11‐RAD50‐NBS1
mRNA‐Seq: high‐throughput mRNA sequencing
MS: mass spectrometry
NER: nucleotide excision repair
NES: nuclear export signal
NLS: nuclear localization signal
PTM: post‐translational modification
TCGA: The Cancer Genome Atlas
UV: ultraviolet
VM: vascular mimicry
WT: wild type

## 1 Introduction

DICER, a member of the RNase III family, is a nucleic acid endonuclease whose main function is to process precursor microRNAs into mature miRNAs [1]. Low expression or dysfunction of DICER has been associated with low‐microRNA (miRNA) levels in many human cancers [2, 3, 4, 5]. However, there is increasing evidence that DICER is localized not only in the cytoplasm for miRNA synthesis but also in the nucleus for a variety of biological processes such as chromatin remodelling, epigenetic modification and DNA damage repair [6, 7, 8, 9, 10]. The DICER expression is finely regulated at both transcriptional and post‐transcriptional levels, and its abnormal expression can lead to cell dysfunction and the occurrence of diseases including tumours [11]. The transcription factor Sox4 [12] and microphthalmia‐associated transcription factor (MITF) [13] up‐regulate DICER expression at the transcription level in melanoma. More importantly, the stability and functions of DICER protein are controlled by many post‐translational modifications (PTMs). For example, SUMOylation of DICER induced by smoking decreases its biological activity and leads to a significant downregulation of miRNA levels in alveolar macrophages [14]. HIF‐1α can recruit the ubiquitin E3 ligase Parkin, which promotes monoubiquitination of DICER and enhances autophagic lysosomal pathway‐mediated protein degradation [15]. The chemotherapeutic agent oxaliplatin induces the ubiquitin E3 ligase CHIP to increase DICER ubiquitination and degradation [16]. ERK phosphorylates the RNase IIIb and double‐stranded RNA (dsRNA)‐binding domains of DICER, resulting in altered localization from the cytoplasm to the nucleus, thereby affecting overall miRNA production [17]. DICER is also subject to glycosylation modifications, which may contribute to its normal folding and functions [18].

Growing evidence demonstrates that DICER in the nucleus is involved in DNA damage repair. Upon DNA damage DICER is phosphorylated and then accumulates in the nucleus [19, 20]. Phosphorylated DICER is recruited to DNA double‐strand breaks (DSBs), to process nascent RNA into a small noncoding DNA‐damage‐response RNA (DDRNA), which in turn participates in subsequent signalling [21, 22, 23, 24, 25]. DDRNA is essential for the recruitment of the secondary repair factors MDC1 and 53BP1 for DNA damage repair, but not necessary for the recruitment of the primary repair factors such as the MRE11‐RAD50‐NBS1 (MRN) complex [21]. Thus, DICER, a key enzyme for DDRNA processing, has a very important position in DSB repair signalling. Upon UV‐induced DNA damage, there is also a DDRNA process mediated by DICER similar to that of DSB [26]. Small DDRNAs (such as siRNAs or miRNAs) generated by DICER are recruited to ARGONAUTE proteins (AGOs), forming a chromatin‐bound complex with other proteins such as DDB2, which facilitates a recognition of DNA damages in an RNA/DNA complementary strand‐specific manner at damaged sites and the next step of DNA repair [26, 27]. In addition, DICER together with ZRF1 is implicated in chromatin decondensation during nucleotide excision repair (NER) upon UV irradiation [28].

USP7, also named as herpes‐associated USP (HAUSP), is one of the most well‐investigated deubiquitinases [29, 30]. USP7 plays important roles in epigenetic regulation, cell signalling, DNA damage repair, immunological responses and tumourigenesis by regulating the localization, activation and stability of its substrates via deubiquitination. USP7 can regulate the stability of many proteins, such as p53 [31], MDM2 [32], N‐MYC [33], UHRF1 [34], PHF8 [35], NOTCH1 [36, 37] and DNMT1 [38], it also affects the localization of FOXO4 [39] and PTEN [40] by removing a single ubiquitin molecule. USP7 is overexpressed in a variety of human cancers, thus it has gradually become a new target in cancer therapy [41, 42] and small molecule inhibitors for USP7 are being developed [43, 44, 45, 46]. Oncoprotein MDM2 is also highly expressed in many human cancers [47], including colorectal adenocarcinoma, breast carcinoma, and lung cancer. MDM2 is considered a potential target for cancer therapy because of its negative regulatory role on p53 [48, 49].

In this study, we found that USP7 bound to DICER but surprisingly down‐regulated its protein level in the nucleus, which impacted DNA damage response (DDR) and cancer progression. MDM2, as a substrate of USP7, was identified as a novel DICER ubiquitin E3 ligase, thus forming a new regulatory axis of USP7‐MDM2‐DICER. Our research discovered a novel mechanism in the regulation of the expression level and function of nucleic DICER.

## 2 Materials and methods

### 2.1 Cell culture and transfection

HEK293T (RRID: CVCL_0063), HEK293FT (RRID: CVCL_6911), HepG2 (RRID: CVCL_0027) and DU145 (RRID: CVCL_0105) cell lines were cultured in Dulbecco's modified Eagle's medium (Corning, NY, USA) containing 10% FBS (YEASEN, Shanghai, China) and 1% penicillin/streptomycin (YEASEN) at 37 °C with 5% CO_2_. MDA‐MB‐231 (RRID: CVCL_0062), MDA‐MB‐453 (RRID: CVCL_0418), MDA‐MB‐468 (RRID: CVCL_0419) cell lines were cultured in Leibovitz's L‐15 medium (Hyclone, Logan, UT, USA) containing 10% FBS and 1% penicillin/streptomycin at 37 °C without CO_2_. T47D (RRID: CVCL_0553), HCC1937 (RRID: CVCL_0290), SKBR3 (RRID: CVCL_0033), and BT549 (RRID: CVCL_1092) cell lines were cultured in RPMI‐1640 medium (Corning) containing 10% FBS and 1% penicillin/streptomycin at 37 °C with 5% CO_2_. Transfections were performed by using Lipofectamine 2000 (Invitrogen, Carlsbad, CA, USA). The breast cell lines are kind gifts from Prof. Qian Zhao (Shanghai Jiao Tong University School of Medicine, Shanghai, China). The other cell lines were from Cell Bank/Stem Cell Bank, Chinese Academy of Sciences. All cell lines have been authenticated in the past 3 years by Short Tandem Repeat (STR) analysis. Experiments were performed in mycoplasma‐free cells.

### 2.2 Antibodies and reagents

Antibodies against DICER (#3363), β‐tubulin (#2146) and γ‐H2AX (#9718) were purchased from Cell Signaling Technology (Danvers, MA, USA). Antibody against USP7 (#A300‐033A) was purchased from BETHYL (Montgomery, TX, USA). Antibodies against GAPDH (#60004‐1‐Ig) and β‐actin (#60008‐1‐Ig) were purchased from Proteintech Group (Rosemont, IL, USA). Antibodies against MDM2 (#sc‐965), Ubiquitin (#sc‐8017), XPF (#sc‐136 153), normal mouse IgG (#sc‐2025) and normal rabbit IgG (#sc‐2027) were purchased from Santa Cruz Biotechnology (Santa Cruz, CA, USA). Antibody against Flag‐tag (#F1804) was purchased from Sigma‐Aldrich (St. Louis, MO, USA). Antibody against HA‐tag (#MMS‐101R) was purchased from Covance (Berkeley, CA, USA). Antibody against 53BP1 (#NB100‐304) was purchased from Novus Biologicals (Centennial, CO, USA). ProteinG Plus/ProteinA agarose suspension (#IP05) was purchased from Calbiochem (San Diego, CA, USA). Polybrene (#H9268), cycloheximide (#C7698) and puromycin (#P8833) were purchased from Sigma‐Aldrich. Camptothecin (CPT, #S1288) was purchased from Selleck (Houston, TX, USA).

### 2.3 Plasmid constructions

The expression plasmid HA‐Flag‐DICER (kindly provided by V. Narry Kim at the Seoul National University) was mutated to a catalytically inactive mutant (D1320A and D1709A) according to the previous study [1]. The plasmid Flag‐USP7 was subcloned into pCMV‐Flag and then mutated to a catalytically inactive mutant (C223S). The plasmid HA‐MDM2 was subcloned into pCMV‐HA and then mutated to a catalytically inactive mutant (C464A). HA‐MDM2‐NES‐Mut and HA‐MDM2‐NLS‐Mut plasmids were constructed according to the literature [50]. The shRNA oligonucleotides for USP7, MDM2 and DICER referred from Sigma were subcloned into the lentiviral vector pLKO.1. Primer sequences for plasmid constructions, point mutations and shRNAs were listed (Table S1).

### 2.4 qRT–PCR

RNA was extracted by TRIZOL reagent (Sigma‐Aldrich) and then treated with DNase I (ThermoFisher, Mississauga, Canada) to degrade genomic DNA. Reverse transcription was performed by using the PrimeScript RT–PCR Kit (#RR037A, TAKARA, Otsu, Shiga, Japan) according to the manufacturer's instructions. qRT–PCR was performed with SYBR Green PCR Master Mix (#4309155, Applied Biosystems, Waltham, MA, USA) to analyze the RNA abundance of indicated mRNAs. Primers used for qRT–PCR are listed in Table S1.

### 2.5 Co‐immunoprecipitation (Co‐IP)

In total, 48–72 h (hours) after transfection, 293T cells were lysed with ice‐cold RIPA buffer (50 mm Tris–HCl, 150 mm NaCl, 1% NP‐40, protease inhibitor cocktail). Cell lysates were incubated with Protein A/G agarose beads and antibodies at 4 °C overnight, followed by 3–5 times washing with RIPA buffer, and then analyzed by Western blotting (WB).

### 2.6 Immunofluorescence (IF) staining

For regular IF staining, cells were seeded on the glass cover slips in 24‐well plates, treated according to the requirements, and fixed with 4% paraformaldehyde for 30 min at room temperature. After washing with PBS 3 times (5 min each time), cells were blocked in blocking solution (TBS with 5% BSA and 0.5% Triton X‐100) for 1 h and then incubated with indicated antibodies at 4 °C overnight. After washing with PBS, cells were incubated with appropriate secondary antibodies conjugated with AlexaFluorescence 488 or 568 in blocking solution for 1 h at room temperature away from light, and then the nucleus was stained by DAPI for 30 min in the dark. After washing with PBS and treating with antifade mountant (Thermo Fisher), the IF images were recorded by laser scanning confocal microscopy.

For IF staining of DICER in the nuclei, to exclude cytoplasmic DICER and maintain nuclear DICER, cells were treated with 0.1% Triton X‐100 on ice for 5 min before being fixed with 4% paraformaldehyde. Subsequent steps are the same as above.

### 2.7 Mass spectrometry (MS) analysis

Immunoprecipitates pulled down with anti‐Flag antibody or anti‐IgG from 293T cells transfected with HA‐Flag‐DICER plasmid were collected by SDS–PAGE. The proteomic analysis based on LC–MS/MS was performed at the Proteomics of Core Facility of Basic Medical Sciences, Shanghai Jiao Tong University School of Medicine. Raw data were processed with MaxQuant and searched against the *Homo sapiens* Uniprot database using the Andromeda search engine integrated into MaxQuant and default settings were applied.

### 2.8 Cell viability

The cell relative viability under DNA damage conditions was measured by Cell Counting Kit‐8 assay. Stable cell lines were seeded at a density of 1 × 10^4^ on 96‐well plates. The day after plating, cells were treated with the specified intensity of UV exposure and CPT concentration. After 48 h, the cellular activity was tested by adding 10 μL of CCK8 reagents into each well and reading the absorbance at a wavelength of 450 nm. The relative viability of cells was measured by the ratio of absorbance under DNA damage to normal conditions.

### 2.9 Plate colony formation assay

Cell colony formation ability under DNA damage conditions was measured as follows. Stable cell lines were seeded at a density of 500 cells on 12‐well plates. Cells were treated with the specified intensity of UV exposure and Camptothecin (CPT) concentration. After 7–10 days, colonies were stained with 0.1% crystal violet overnight, and the number of colonies was counted by Image J.

### 2.10 Soft agar colony formation assay

The soft agar colony formation assay was performed as previously described [51]. 2 mL base gel (2 × medium, 10% FBS, 1% penicillin/streptomycin and 0.6% agar gel) was placed in six‐well plates, and then followed by layering 1 × 10^3^ or 2 × 10^3^ cells in 2 mL of colony formation gel (2 × medium, 10% FBS, 1% penicillin/streptomycin and 0.35% agar gel) on the top of base gel. After 3–4 weeks, gels were stained with 0.005% crystal violet overnight, and the number of colonies was counted by Image J.

### 2.11 Wound‐healing assay

For the wound‐healing assay, firstly prepare the culture‐inserts (IBIDI) on a flat and clean surface. Seed the cells and wait for cell attachment according to the manufacturer's instructions. Remove the culture‐inserts and fill with serum‐free or low‐serum (1% FBS) medium. Photos were taken as indicated time until the wound was healed.

### 2.12 Vasculogenic mimicry

The vasculogenic mimicry experiment of stable cell lines was carried out using μ‐Slide (IBIDI) according to the manufacturer's protocol. 10 μL of 3D matrix gel (Millipore, Oakville, Canada) were added to the wells of μ‐slide, and 5000 cells were seeded on the top of the gel. Pictures were taken with a microscope 12 h later.

### 2.13 3D culture growth assay

The 3D cell culture was carried out according to the methods described before [52]. Mixtures of 5 μL of cell sUSPension (500 cells) and 5 μL of 3D matrix gel (Millipore) were added to the wells of μ‐Slide (IBIDI), covered with complete medium, and cultured for 5–7 days. Colonies were observed and recorded by microscope.

### 2.14 High‐throughput sequencing for miRNA‐Seq and RNA‐Seq

For miRNA‐Seq, total RNA was extracted from stable cell lines by using a TRIZOL reagent. Extracted RNA was used for preparing the miRNA sequencing library, which included 3′‐adaptor ligation, 5′‐adaptor ligation, cDNA synthesis and PCR amplification and size selection of ~ 150 bp PCR amplicons (corresponding to ~ 22 nt miRNAs). The libraries were denatured as single‐stranded DNA molecules, captured on Illumina flow cells, amplified *in situ* as clusters and finally sequenced for 50 cycles on Illumina HiSeq sequencer following the manufacturer's instructions.

For RNA‐Seq, 150 bp paired‐end reads were harvested from Illumina HiSeq 4000 sequencer and were quality controlled by Q30. After 3′ adaptor‐trimming and low‐quality reads removing by cutadapt software (v1.9.3). The high‐quality reads were aligned to the human reference genome (UCSC hg19) with hisat2 software (v2.0.4). Then, guided by the Ensembl gtf gene annotation file, cuffdiff software (v2.2.1, part of cufflinks) was used to get the FPKM as the expression profile of mRNAs. For the identification of differentially expressed genes (DEGs), the expression profile of shUSP7 was compared to that of shCtrl, and the expression profile of shUSP7shDICER was compared to that of shUSP7. For MDA‐MB‐231 with duplicate samples, DEGs were identified by FPKM ≥ 1, Fold change ≥ 1.5 and *P* value ≤ 0.05. For DU145 with a single sample, DEGs were identified by FPKM ≥ 1 and Fold change ≥ 1.5. High‐throughput sequencing for miRNA‐Seq and RNA‐Seq were performed by Cloud‐Seq Biotech (Shanghai, China).

### 2.15 GSEA and GSVA analysis

GSEA analysis was performed with gsea software 4.0 [53]. GSVA analysis was performed with r package gsva [54].

### 2.16 Xenograft tumour model

All the animal experiments were conducted in agreement with the Guide for the Care and Use of Laboratory Animals and approved by the Institutional Animal Care and Use Committee of Shanghai Jiao Tong University School of Medicine (Approval NO. A‐2019‐036). The mice were purchased from Shanghai Lingchang Biotechnology Co., Ltd (Shanghai, China), housing in a specific pathogen‐free environment, handled with care and allowing for adaption to the environment before experiments. The experiment of the xenograft tumour model was established as described previously [55]. Stable DU145 cell lines were injected subcutaneously into 6‐week‐old male BALB/c nude mice, 2.5 × 10^6^ cells for each mouse, *n* = 5. Mice were sacrificed 3 weeks later, and tumours were weighed and photographed.

### 2.17 Statistical analysis

Statistical analyses were performed with graphpad prism 8 (Graphpad Software, Boston, MA, USA). All data are presented as the means ± SD for qPCR, soft agar assay, mouse xenograft model. The Spearman correlation analysis was performed to analyze the association between the expressions of DICER with USP7 and MDM2 expression levels. Comparisons between groups for statistical significance were conducted with a 2‐tailed‐unpaired Student's *t*‐test. *P* ≤ 0.05(*), *P* ≤ 0.01(**) and *P* ≤ 0.001(***) were considered statistically significant.

## 3 Results

### 3.1 USP7 interacts with and downregulates DICER

**Fig. 1:**
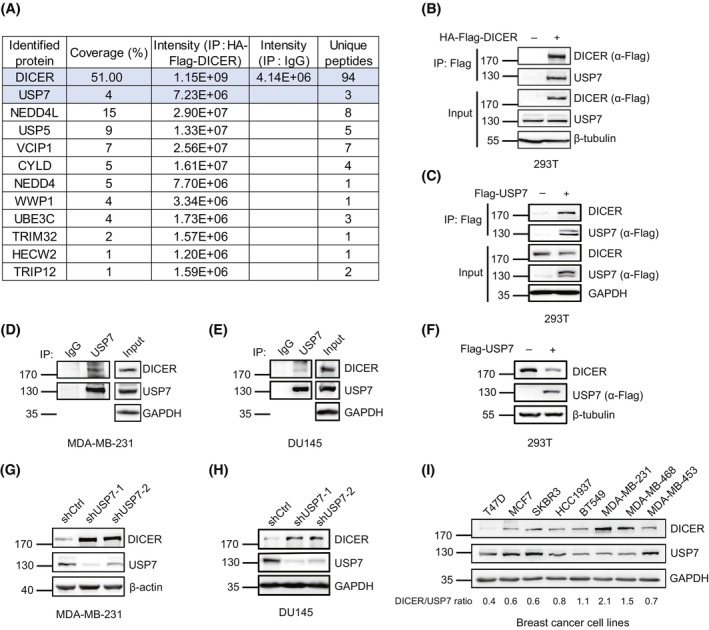
USP7 interacts with and downregulates DICER protein. (A) Co‐immunoprecipitates from 293T cells expressing HA‐Flag‐DICER were subjected to mass spectrometry analysis, *n* = 1. A number of DICER‐interacting proteins related to ubiquitination or deubiquitination were listed. (B, C) Co‐immunoprecipitation (Co‐IP) showing the interaction of HA‐Flag‐DICER and endogenous USP7 (B), or Flag‐USP7 and endogenous DICER (C) in 293T cells. (D, E) Co‐IP showing the interaction of endogenous DICER and USP7 in MDA‐MB‐231 (D) and DU145 (E) cells. (F) The DICER protein level in 293T cells overexpressing Flag‐USP7 was detected by Western blot (WB). (G, H) Endogenous USP7 in MDA‐MB‐231 (G) and DU145 (H) cells was knocked down by two specific shRNAs, and then DICER was detected by WB. (I) WB showing the expression levels of DICER and USP7 in different breast cancer cell lines. WB results were all shown with one representative image from three independent experiments.

To further investigate the potential interacting proteins of DICER, we expressed HA‐Flag‐DICER in 293T cells and performed Co‐IP for MS analysis (Table S2), showing more than 10 proteins related to ubiquitination (Fig. 1A). One of these proteins, USP7 has also been identified as an DICER‐interacting protein in 293T cells by MS [28, 56], so we speculated that USP7 may be a potential regulator of DICER. To verify this, lysates from 293T cells transfected with HA‐Flag‐DICER or Flag‐USP7 plasmid were used for Co‐IP with anti‐Flag antibody and followed by immunoblotting, showing that DICER indeed interacted with USP7 (Fig. 1B,C). More convincingly, the interaction of endogenous USP7 and DICER was confirmed in human breast cancer cell lines MDA‐MB‐231 (Fig. 1D) and HCC1937 (Fig. S1A), prostate cancer cell line DU145 (Fig. 1E), and liver cancer cell line HepG2 (Fig. S1B).

Since USP7 interacted with DICER, we speculated that USP7 might function as a deubiquitination enzyme to stabilize DICER. Unexpectedly, when Flag‐USP7 was overexpressed in 293T cells, endogenous DICER protein was significantly decreased but not stabilized (Fig. 1F). In accordance with this, DICER protein was significantly increased when USP7 was stably knocked down by specific shRNAs in serval cell lines MDA‐MB‐231 (Fig. 1G), DU145 (Fig. 1H), PC3 (Fig. S1C), MDA‐MB‐468 (Fig. S1D), HepG2 (Fig. S1E) and H1299 (Fig. S1F). Interestingly, the levels of DICER protein were significantly negatively correlated with those of USP7 in different breast cancer cells (Fig. 1I). These results suggest that USP7 was a negative regulator rather than a deubiquitinating enzyme of DICER.

Further to determine how USP7 regulates DICER, we first detected the mRNA level of DICER in USP7‐knockdown MDA‐MB‐231 and DU145 cells by qRT–PCR, showing that there was no significant difference in the expression of DICER mRNA between USP7‐knockdown group and control group (Fig. S1G,H). In 293T cells transfected with Flag‐USP7 and HA‐Flag‐DICER, we found that the treatment with a proteasome inhibitor MG132 significantly inhibited DICER degradation mediated by USP7 (Fig. S1I). These indicated that the downregulation of DICER by USP7 was at the protein level rather than the transcription level. Moreover, DICER protein was greatly reduced by overexpression of wild‐type (WT) USP7 but not by the catalytically inactive mutant C223S of USP7 in 293T cells (Fig. S1J), indicating that the negative regulation of USP7 on DICER required its enzyme activity.

### 3.2 Knockdown of USP7 inhibits cancer progression by upregulation of DICER

**Fig. 2:**
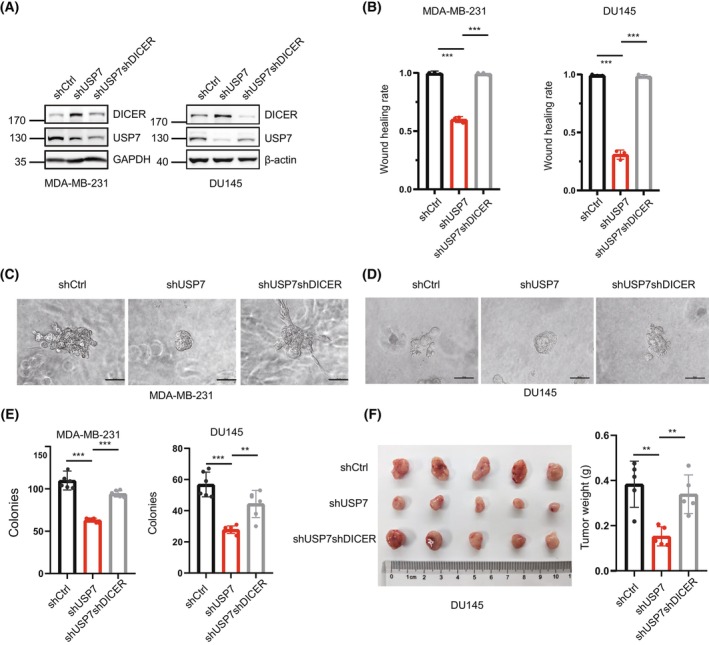
Knockdown of USP7 inhibits cancer progression by upregulation of DICER. (A) Construction of stable cell lines by knocking down DICER with shRNA in USP7‐knockdown MDA‐MB‐231 and DU145 cells. WB results were shown with one representative image from three independent experiments. (B) The quantification results of wound healing assay in stable MDA‐MB‐231 and DU145 cell lines, *n* = 3. (C, D) 3D culture assay in MDA‐MB‐231 (C) and DU145 (D) cells, photographs were taken 5 days later, scale: 50 μm, *n* = 3. (E) Statistics of colonies number in the soft‐agar colony formation assay, MDA‐MB‐231 cells were seeded at a density of 2000/well and cultured for 4 weeks, DU145 cells were seeded at a density of 1000/well and cultured for 3 weeks, *n* = 6. (F) Stable DU145 cell lines were subcutaneously injected into 6‐week‐old male BALB/c nude mice individually (2.5 × 10^6^ cells per each), *n* = 5. Mice were sacrificed 3 weeks later and the tumours were dissected. Data are presented as the mean ± SD. *P* ≤ 0.05(*), *P* ≤ 0.01(**) and *P* ≤ 0.001(***), as determined by 2‐tailed‐unpaired Student's *t*‐test.

USP7 is an oncoprotein and is highly expressed in various human cancers [35, 41, 57, 58, 59]. Since we found that knockdown of USP7 by shRNA (shUSP7) led to a significant increase in the protein level of DICER, and DICER usually acts as a tumour suppressor according to several studies [2, 3, 5, 60], it is possible that USP7 plays a role in cancer progression through DICER. Thus, we knocked down DICER in stable MDA‐MB‐231‐shUSP7 and DU145‐shUSP7 cell lines (Fig. 2A) to observe whether shUSP7shDICER can reverse the phenotypic changes induced by shUSP7. Firstly, the tubular structure formation of vascular mimicry (VM) was greatly weakened in the shUSP7 group, which was almost recovered in the shUSP7shDICER group in both MDA‐MB‐231 and DU145 cells, suggesting that USP7 regulated the ability of vascular mimicry formation of tumour cells at least partially through DICER (Fig. S2A). The wound‐healing assays were conducted to evaluate the effects of USP7‐DICER on migration, showing that the cell migratory capacity was drastically decreased in the shUSP7 group whereas recovered by shUSP7shDICER group (Fig. 2B, Fig. S2B,C). The 3D culture growth assays showed that shUSP7 cells formed a smooth clone with fewer burr such as protrusions around the clone edge, while shUSP7shDICER cells were similar to the shCtrl group, with scattered and invasive morphology (Fig. 2C,D). In consistent with these results, the number of soft‐agar colonies formed in the shUSP7 group was significantly decreased whereas increased in the shUSP7shDICER group (Fig. 2E). To further verify whether USP7 regulates cancer progression through DICER *in vivo*, xenografted tumour growth analysis was performed. The above DU145 stable cell lines were subcutaneously injected into the back of male BALB/c nude mice. The nude mice were sacrificed at 3 weeks after injection, tumours were photographed (Fig. 2F, left panel) and their weights were analyzed (Fig. 2F, right panel), showing that the shUSP7 group had smaller tumours while the shUSP7shDICER group almost completely reverse the tumour‐suppressing effect of USP7 knockdown *in vivo*. Taken together, the above results suggested that USP7‐DICER formed a new regulatory axis and played a regulatory role in cancer progression.

### 3.3 USP7‐DICER axis regulates the mRNA expression profile

Since the main function of DICER is to participate in the miRNA synthesis in the cytoplasm, we speculate that the USP7‐DICER axis may play a role by regulating miRNAs. Therefore, we firstly conducted the high‐throughput miRNA sequencing (miRNA‐Seq) by using the above DU145 and MDA‐MB‐231 stable cell lines. Unexpectedly, the comparison of miRNA expression profiles showed that there was no significant difference between shCtrl, shUSP7 and shUSP7shDICER groups in either DU145 or MDA‐MB‐231 cell lines (Fig. S3A,B), which suggested that the increased DICER protein by USP7 knockdown did not affect the overall miRNA expression level.

**Fig. 3:**
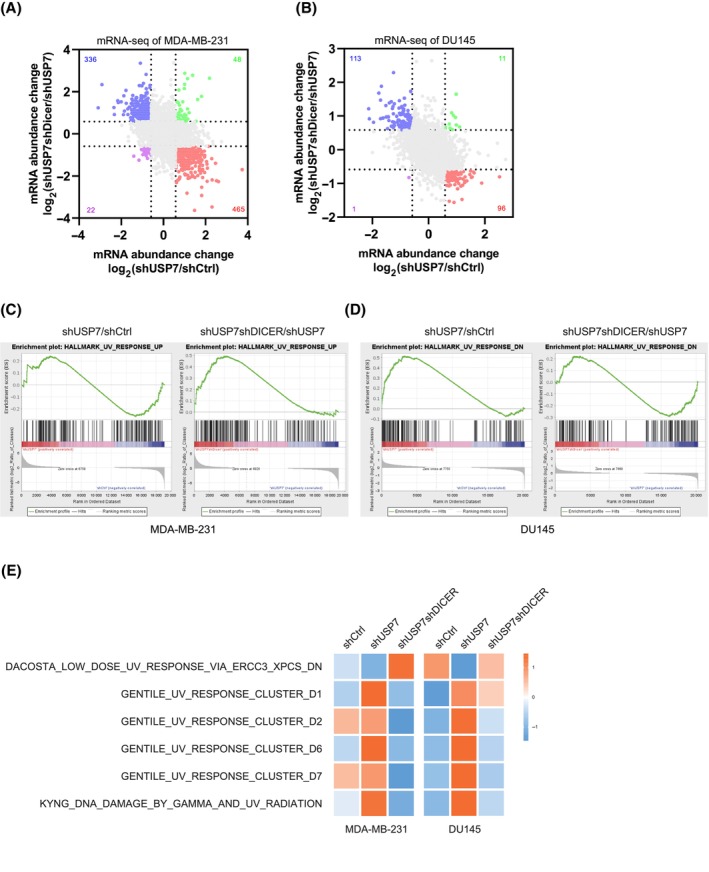
USP7‐DICER axis regulates mRNA expression profile. (A, B) Scatter plots showing the mRNA expression changes in MDA‐MB‐231 (A) and DU145 (B) stable cell lines, 1 replicate for DU145 cell samples and 2 replicates for MDA‐MB‐231 cell samples. (C, D) Gene Set Enrichment Analysis (GSEA) of mRNA‐Seq results were enriched into UV response related gene set in MDA‐MB‐231 (C) and DU145 (D) stable cell lines. (E) Gene Set Variation Analysis (GSVA) analysis of MDA‐MB‐231 (mean value of two samples) and DU145 stable cell lines based on UV‐related gene sets.

Nevertheless, we performed the high‐throughput sequencing of mRNA (mRNA‐Seq) to show that the shUSP7shDICER group reversed the mRNA expression changes in the shUSP7 group (Fig. 3A,B and Fig. S3C,D), implying that the expression levels of some mRNAs were obviously regulated by the USP7‐DICER axis. By using the gene ontology (GO) and the Kyoto Encyclopedia of Genes and Genomes (KEGG) database, we analyzed the differentially expressed genes regulated by the USP7‐DICER axis but failed to find common pathways in both MDA‐MB‐231 and DU145 (Fig. S3E,F). However, we conducted Gene Set Enrichment Analysis (GSEA) and interestingly, found that the mRNA expression changes in both DU145 and MDA‐MB‐231 cell lines were significantly enriched in a gene set related to ultraviolet (UV) light response (Fig. 3C,D). Furthermore, some other UV‐related gene sets were also enriched by Gene Set Variation Analysis (GSVA) (Fig. 3E). As known that UV stimulation can cause DNA damage in cells and abnormal repair of DNA damage causes tumourigenesis, thus we hypothesized that the USP7‐DICER axis regulating cancer occurrence and development may be involved in DNA damage repair.

### 3.4 USP7‐DICER axis plays an important role in DNA damage repair

**Fig. 4:**
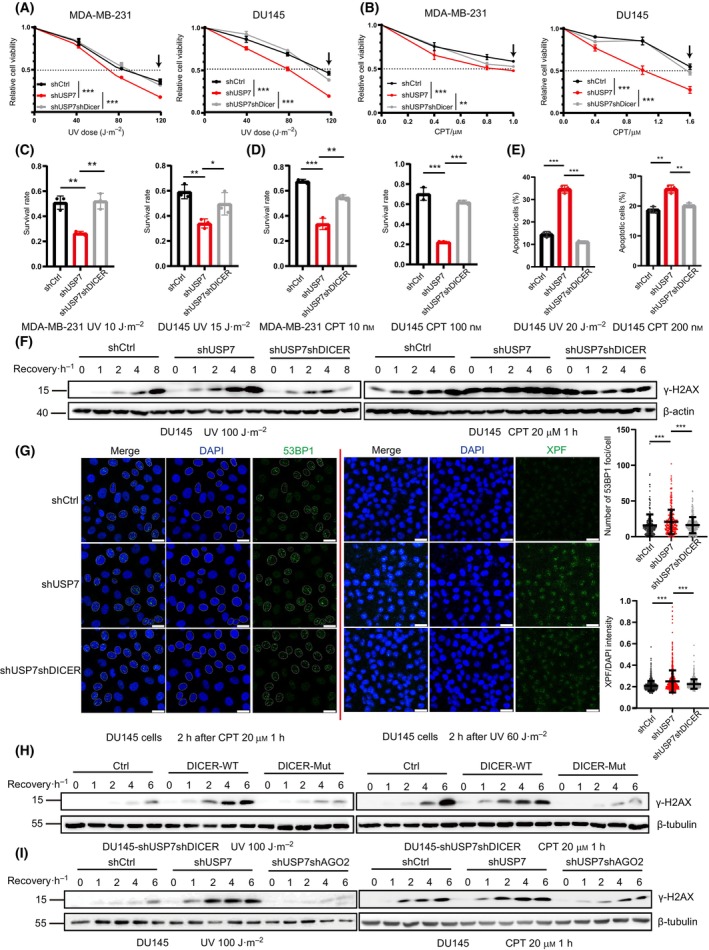
USP7‐DICER axis regulates DNA damage response. (A, B) The relative cell viability was measured by using CCK8 reagent under indicated UV dose (A) or Camptothecin (CPT) concentration (B) treatment for MDA‐MB‐231 and DU145 stable cell lines, *n* = 4, *P*‐values indicated the significant difference in cell viability between the last dosage group (with arrow indicated) for the three cell groups, the dashed line indicated the 50% relative cell viability. (C, D) Survival rate under UV (C) or CPT (D) treatments compared with untreated group for MDA‐MB‐231 and DU145 stable cell lines, *n* = 3. (E) Apoptosis rate of cells treated with UV and CPT were analyzed by flow cytometry, *n* = 3. (F) The levels of γ‐H2AX in DU145 stable cell lines in different time after stimulation with 100 J·m^−2^ UV or 20 μm CPT. (G) Cells were treated with 20 μm CPT or 60 J·m^−2^ UV, fixed 2 h later, and the foci number of 53BP1 and relative intensity of XPF was observed by immunofluorescence, *n* > 200, scale bar: 25 μm. (H) The effect of DICER–WT and DICER‐Mut on γ‐H2AX signal in DU145 stable cell lines in different time after stimulation with 100 J·m^−2^ UV or 20 μm CPT. (I) The effect of AGO2 knockdown on γ‐H2AX signal in DU145 stable cell lines in different time after stimulation with 100 J·m^−2^ UV or 20 μm CPT. Data are presented as the mean ± SD. *P* ≤ 0.05(*), *P* ≤ 0.01(**) and *P* ≤ 0.001(***), as determined by 2‐tailed‐unpaired Student's *t*‐test. Western blot results were all shown with one representative image from three independent experiments.

To determine whether the USP7‐DICER axis functions in response to DNA damage, above MDA‐MB‐231 and DU145 stable cell lines were stimulated by different doses of UV to observe changes in cell viability. The cell viability in the shUSP7 group was significantly decreased when compared with the shCtrl group, whereas the shUSP7shDICER group reversed this trend (Fig. 4A). Camptothecine (CPT), an inhibitor of topoisomerase I, is a common antitumour chemotherapeutic agent and usually causes a replication fork block where is highly susceptible to DNA double‐strand breaks. To confirm whether the USP7‐DICER axis is involved in DNA damage repair, cells were also treated with CPT. The results of cell viability assays with CPT were consistent with the UV treatment results (Fig. 4B). We further conducted the plate colony formation experiments by treating MDA‐MB‐231 and DU145 stable cell lines with UV and CPT. The results showed that the colony survival rate of the shUSP7 group was lower than that of the shCtrl group, while that of the shUSP7shDICER group was restored (Fig. 4C,D and Fig. S4A,B). The transcription factor p53 is a deubiquitinated substrate of USP7 and has a pivotal role in the DNA damage repair process. Earlier studies demonstrate that WT p53 normally causes cell cycle arrest in the G1 phase under a variety of DNA damage stimulation conditions, thus promoting cell death [61, 62]. Therefore, we wanted to test whether the regulation of the USP7‐DICER axis on DNA damage repair‐related phenotypes is dependent on p53. For a valid comparison, two lung cancer cell lines, A549 (p53‐WT) and H1299 (p53‐Null) were chosen for the construction of stable cell lines shCtrl, shUSP7 and shUSP7shDICER (Fig. S4C) with the same strategy as above. These stable cell lines were employed in the CPT‐treated plate colony formation experiment to show consistent results between two kinds of cell lines A549 and H1299 (Fig. S4D,E), indicating that the regulation of DNA damage repair by the USP7‐DICER axis was independent of p53.

Since the above data showed the USP7‐DICER axis significantly affecting cell viability and colony formation number, which may be caused by apoptosis, we measured the apoptosis ratios of DU145 stable cell lines under UV and CPT treatments. The results showed that the apoptosis rate of the shUSP7 group was very significantly increased, while that of the shUSP7shDICER group was decreased (Fig. 4E, Fig. S4F). This indicated that the USP7‐DICER axis regulated cell apoptosis, which contributed to the phenotype of cell viability and clonal survival under DNA damage stresses.

To more confirm the USP7‐DICER axis in the regulation of DNA damage repair, the intensity of γ‐H2AX which is the most representative DDR signal [63, 64, 65] was detected. DU145 stable cell lines were treated with UV (100 J·m^−2^) and CPT (20 μm), respectively, and then cells were collected at different time points after treatments. The results showed that the γ‐H2AX level in the shUSP7 group was higher than that in the shCtrl group, while the γ‐H2AX level in the shUSP7shDICER group was restored to similar levels as that of the shCtrl group, demonstrating that the USP7‐DICER axis regulated DNA damage repair (Fig. 4F, Fig. S4G). In addition to the marker γ‐H2AX, 53BP1 and XPF are often used as landmark repair signals after CPT and UV treatment, respectively [66]. Thus, immunofluorescence (IF) staining was performed to observe the number of 53BP1 foci in CPT‐treated nuclei, and the number of foci in each nucleus was counted using the focus quantification algorithm FoCo [67]. The results showed that the number of 53BP1 foci in the shUSP7 group was significantly increased compared with the shCtrl group, while the number in the shUSP7shDICER group was recovered to a similar level as that of the shCtrl group (Fig. 4G). The fluorescence intensity of XPF by IF staining was also observed after UV treatment. The fluorescence intensities of DAPI and XPF in each nucleus were counted by using the software cellprofiler [68] to analyze the differences in the relative fluorescence intensity of XPF/DAPI in several groups. Consistently, the results showed that the relative fluorescence intensity of XPF in the shUSP7 group was significantly increased compared to that in the shCtrl group, while that in the shUSP7shDICER group was recovered to a similar intensity as that of shCtrl (Fig. 4G). The above results demonstrated that the USP7‐DICER axis played an important role in DNA damage repair. However, the effects of USP7 knockdown on γ‐H2AX and 53BP1 remain controversial. We found that knocking down USP7 caused elevated γ‐ H2AX levels and 53BP1 lesion formation, which is consistent with the conclusion of several previous studies [69, 70, 71], although there are opposite conclusions [72, 73]. This may be due to the involvement of USP7 in multiple steps of DNA damage repair, resulting in more complex functions; and different experimental conditions in different studies may also be one of the reasons.

As reported, upon UV‐mediated damage or double‐strand break (DSB) damage, DICER can act as an RNase III endonuclease to generate DDRNA, which binds to AGO2 at the DNA damage site for downstream repair factor recruitment and signalling transduction [26, 27]. Moreover, DICER can also recruit a methyltransferase MMSET to the damage site in an enzyme activity‐independent manner, and affect the damage signal by influencing epigenetic modification [74]. To explore how the USP7‐DICER axis affects DNA damage repair, we first wanted to determine whether the enzymatic activity of DICER is required. We generated a mutant DICER‐Mut containing the double mutation of D1320A and D1709A in its enzymatic activation domains Rnase IIIA and IIIB [1], respectively. Re‐expression of DICER‐WT in shUSP7shDICER cells showed a strong effect on γ‐H2AX signaling whereas re‐expression of DICER‐Mut had no similar effect as similar with control‐vector group after UV or CPT treatment (Fig. 4H, Fig. S4H). These indicated that the regulation of the USP7‐DICER axis on DNA damage repair was dependent on the endonuclease activity of DICER. To further investigate whether the USP7‐DICER axis is regulated by DDRNA, we constructed a shUSP7shAGO2 cell line to observe whether shAGO2 has similar effects with shDICER. DDRNA processed by DICER binds to the DNA damage site together with AGO2 protein for recruiting downstream repair factors [26]. As expectedly, the results showed that the γ‐H2AX signal was enhanced in the shUSP7 group compared to that in the shCtrl group whereas was weakly responded in the shUSP7shAGO2 group after UV or CPT treatment, which was consistent with the pattern in the shUSP7shDICER group (Fig. 4I, Fig. S4I). Thus, the above results revealed that the function of the USP7‐DICER axis in regulating the DNA damage repair pathway was mediated by DDRNA through AGO2.

### 3.5 DICER mainly accumulates in the nucleus upon knockdown of USP7

**Fig. 5:**
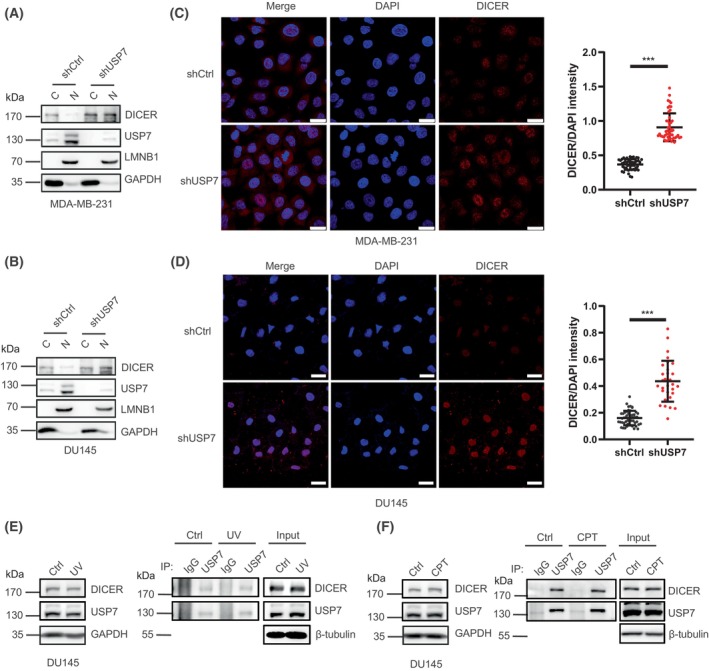
DICER mainly accumulates in the nucleus upon knockdown of USP7. (A–D) Nuclear/Cytosol Fractionation assay (A, B) showed that USP7 negatively regulated DICER protein in the nucleus in MDA‐MB‐231 (A) and DU145 (B) stable cell lines. Immunofluorescence assay (C, D) showed that USP7 negatively regulated DICER in the nucleus in MDA‐MB‐231 (C, left) and DU145 (D, left) stable cell lines, scale bar: 25 μm, the relative intensity of nuclear DICER was quantified from three random views in the right panel of (C) and (D). (E, F) The protein expression levels and interaction between USP7 and DICER were not affected in DU145 cells at 2 h after stimulation with 100 J·m^−2^ UV (E) or 20 μm Camptothecin (CPT) (F). Data are presented as the mean ± SD. *P* ≤ 0.001(***), as determined by 2‐tailed‐unpaired Student's *t*‐test. WB results were all shown with one representative image from three independent experiments.

The above data showed that the DICER protein level was significantly increased by the knockdown of USP7, however, this had no significant effect on the overall miRNA expression level. DICER regulating miRNA synthesis is mainly located in the cytoplasm. But interestingly, we found that the USP7‐DICER axis was involved in the regulation of DNA damage repair in the nucleus. Therefore, we hypothesized that USP7 mainly affects the DICER protein level in the nucleus rather than in the cytoplasm. To validate this, we performed the nucleoplasmic separation experiment on MDA‐MB‐231 and DU145 stable cell lines. Indeed, the increase of DICER protein in the shUSP7 group occurred in the nucleus, not in the cytoplasm (Fig. 5A,B). To further confirm this, we carried out a modified immunofluorescence staining to permeabilize the cells before fixation, so as to ensure that only DICER in the nucleus can be observed without interference from the signal in the cytoplasm. The confocal microscope observation showed that the fluorescence intensity of DICER in shUSP7 nucleus was significantly enhanced compared with shCtrl group (Fig. 5C,D). Additionally, the protein levels of USP7 and DICER and their interactions were not influenced by DNA damage stimulations with UV and CPT (Fig. 5E,F).

### 3.6 MDM2 ubiquitinates DICER to mediate its degradation

**Fig. 6:**
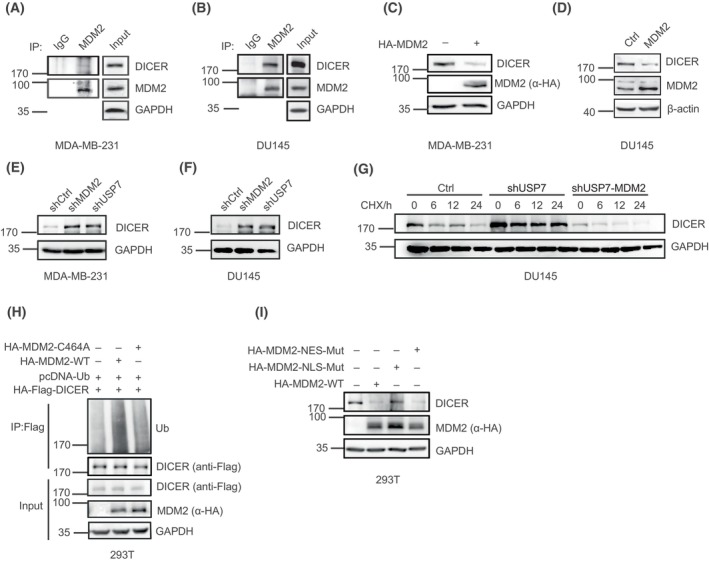
MDM2 ubiquitinates DICER to mediate its degradation. (A, B) The interaction between endogenous MDM2 and DICER was detected in MDA‐MB‐231 (A) or DU145(B) cells by co‐immunoprecipitation (Co‐IP). (C–D) Transiently expressed MDM2 in MDA‐MB‐231 (C) cells, and stably expressed MDM2 in DU145cells (D) resulted in the reduction of endogenous DICER. (E, F) Knockdown of MDM2 led to the increase of DICER in MDA‐MB‐231 (E) and DU145 (F) cells. (G) After 0, 6, 12, 24 h of Cycloheximide (CHX) (100 μg·mL^−1^) treatment, endogenous DICER protein expression level was observed in DU145‐shUSP7‐MDM2, DU145‐shUPS7, and control cell lines, detected by WB. (H) HA‐Flag‐DICER, pcDNA‐Ub and HA‐MDM2‐WT or HA‐MDM2‐C464A (inactive) were overexpressed in 293T cells. Co‐IP with anti‐Flag antibody (DICER) was performed and followed by WBs. (I) HA‐MDM2‐WT, HA‐NLS‐Mut or HA‐NES‐Mut plasmid was transiently expressed in 293T cells, and the protein levels of DICER were detected by WB. WB results were all shown with one representative image from three independent experiments.

Since the mRNA level of DICER was not affected by USP7, there may be a mechanism of USP7 negatively regulating DICER through an ubiquitin E3 ubiquitin ligase. To identify this E3 ligase, we analyzed by combination of known ubiquitin E3 ligases targeted by USP7 [30, 32, 52, 73, 75, 76, 77, 78, 79, 80, 81] (Fig. S5A) and the potential ubiquitin E3 ligases for DICER predicted by using online UbiBrowser (http://ubibrowser.ncpsb.org; Fig. S5B), to find that MDM2 was a candidate E3 ligase for DICER. To verify this, lysates from 293T cells transfected with HA‐MDM2 were used for Co‐IP/WB as indicated, showing exogenously expressed HA‐MDM2 interacting with DICER (Fig. S5C). This interaction was further confirmed by using Co‐IP/WB with endogenous proteins from MDA‐MB‐231 (Fig. 6A) and DU145 (Fig. 6B) cells. To confirm whether MDM2 reduces the DICER protein level by acting as a ubiquitin E3 ligase, we transiently overexpressed HA‐MDM2 in 293T cells and found that the endogenous DICER protein level was indeed decreased (Fig. S5D). By contrast, overexpression of the other two potential E3 ligases NEDD4 and NEDD4L did not affect the DICER protein level (Fig. S5E,F). Consistently with the above results, transiently expressed (Fig. 6C) or stably expressed (Fig. 6D) MDM2 decreased DICER whereas stable knockdown of MDM2 increased DICER in MDA‐MB‐231 (Fig. 6E, Fig. S5G), DU145 (Fig. 6F, Fig. S5H) and HCC1937 cells (Fig. S5I). Moreover, the half‐life of DICER was shorten in stably expressed MDM2 compared to that in the control vector in DU145 cells (Fig. S5J), and DICER became more stable when MDM2 was knocked down in HCC1937 cells (Fig. S5K), indicating that MDM2 as a ubiquitin E3 ligase negatively regulated DICER protein. Interestingly, we found that shMDM2 and shUSP7 caused similar levels of elevated DICER (Fig. 6E,F, Fig. S5G,H), providing more evidence that USP7 is likely to regulate DICER by stabilizing MDM2. Furthermore, we constructed knockdown/overexpression of MDM2 in stable cell lines at shUSP7 conditions (Fig. S5L), showing that MDM2 knockdown led to a further increased DICER protein, while MDM2 overexpression reversed the elevation by shUSP7. The half‐life of DICER in shUSP7‐MDM2 cells was much shorter than that in shUSP7 cells (Fig. 6G, Fig. S5M).

Indeed, overexpression of MDM2‐WT significantly increased the ubiquitination level of DICER while the enzyme‐activated mutant MDM2‐C464A did not in 293T cells (Fig. 6H, Fig. S5N), which confirmed that MDM2 acted as an ubiquitin E3 ligase of DICER. As we have shown USP7 in the regulation of DICER occurs in the nucleus, so next we wanted to validate whether DICER ubiquitination mediated by MDM2 also occurs in the nucleus. MDM2 is a nucleoplasmic protein with conserved sequences of both nuclear export signal (NES) and nuclear localization signal (NLS). We proved that MDM2‐NLS‐Mut, which is mainly located in the cytoplasm, had no effect on the DICER protein level, however both MDM2‐NES‐Mut and MDM2‐WT significantly reduced the DICER protein level (Fig. 6I). Furthermore, we showed that increased DICER by USP7 knockdown resulted from the decrease of MDM2, the substrate of USP7 (Fig. S5O). Therefore, we concluded that the USP7‐MDM2 axis regulated the DICER protein level in the nucleus by the ubiquitin‐proteasome pathway.

### 3.7 USP7‐DICER axis is clinically associated with cancer progression

**Fig. 7:**
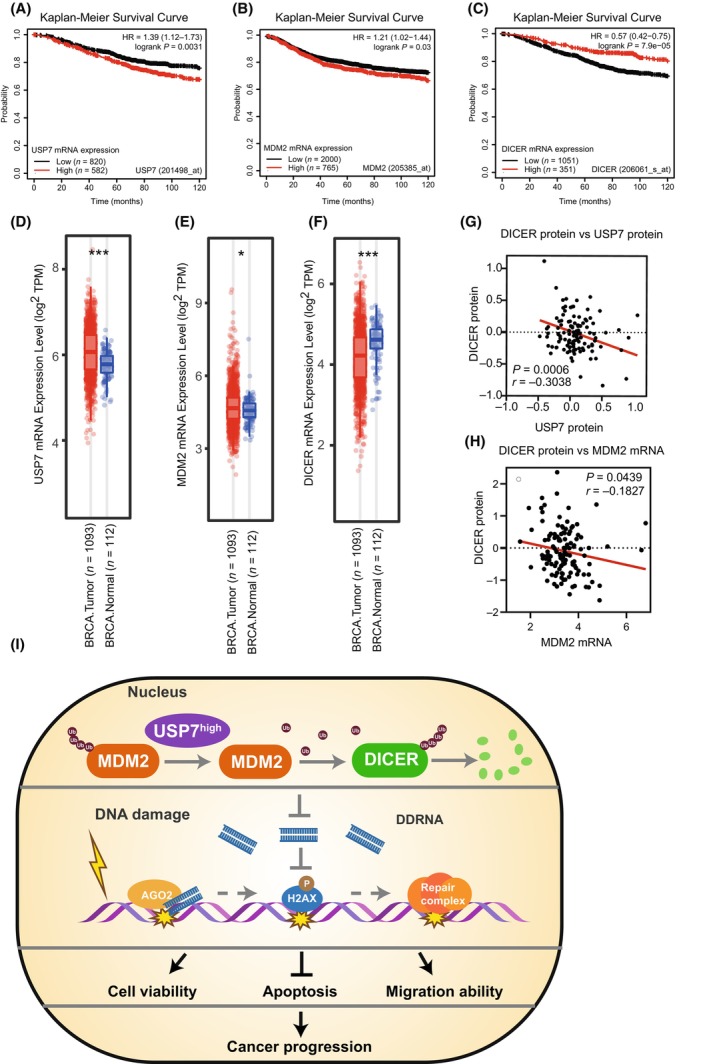
USP7‐DICER axis is clinically associated with cancer progression. (A–C) The Kaplan–Meier analysis of overall patient survival was classified by the mRNA expression level of USP7 (A), MDM2 (B) or DICER (C). The data were obtained from the Kaplan–Meier plotter database. (D–F) The mRNA expression level of USP7 (D), MDM2 (E) or DICER (F) in normal (*n* = 112) and tumour (*n* = 1093) tissues. Data were from patients with breast cancer (BRCA) in The Cancer Genome Atlas (TCGA) database. Data were presented as the mean ± SD in D–F. *P* ≤ 0.05(*), *P* ≤ 0.001(***), as determined by 2‐tailed‐unpaired Student's *t*‐test. (G) Linear regression analysis of the clinical proteomic tumour analysis consortium (CPTAC) database showed a significant negative correlation between DICER and USP7 proteins, *n* = 125, pearson coefficient and *P* values were indicated. (H) Linear regression analysis of the patients with breast cancer showed a significant negative correlation between DICER protein and MDM2 mRNA expression, *n* = 122, spearman coefficient and *P* values were indicated. (I) Model for USP7‐MDM2‐DICER axis in regulation of cancer progression. DDRNA, DNA damage response RNA.

Based on the database of patients with breast cancer (BRCA) from The Cancer Genome Atlas (TCGA), we observed the effect of USP7, MDM2 and DICER mRNA expression levels on the overall survival of patients. The patients with high‐mRNA expression levels of either USP7 (Fig. 7A) or MDM2 (Fig. 7B) had a short survival period and obvious cancer‐promoting characteristics, while patients with high expression levels of DICER (Fig. 7C) had longer survival and cancer suppression characteristics. Furthermore, both USP7 (Fig. 7D) and MDM2 (Fig. 7E) mRNAs were highly expressed in tumour tissues, but DICER (Fig. 7F) mRNA was highly expressed in normal tissues. Convincingly, we verified the negative correlation between the protein levels of USP7 and DICER by using data from the clinical proteomic tumour analysis consortium (CPTAC) (Fig. 7G), which was consistent with our experimental results that USP7 negatively regulated the DICER protein level. Due to the absence of MDM2 protein data, the mRNA data of MDM2 was used for analysis of its correlation with DICER protein. The DICER protein levels were negatively correlated with the MDM2 mRNA levels in patients with BRCA (Fig. 7H), which was consistent with the concept that MDM2 acts as a ubiquitin E3 ligase to mediate DICER degradation.

## 4 Discussion

It is well‐established that DICER has multiple biological functions and is frequently dysregulated in human cancers, but the mechanism of regulating its expression level is still unclear and needs to be explored. In this study, we demonstrated that USP7 negatively regulated DICER *at* the protein level in the nucleus by ubiquitination, which was mediated by MDM2 as a new ubiquitin E3 ligase of DICER, thus forming a new regulatory axis USP7‐MDM2‐DICER. This axis was involved in DNA damage repair through DDRNA processing, which in turn affected cancer progression (Fig. 7I).

The most classical function of DICER is to function in mature miRNA processing in the cytoplasm. Therefore, the regulation of DICER expression level is crucial and PTMs of DICER are well worth studying. DICER is degraded in tumour cells after ubiquitination, and the alteration of the DICER protein level affects the miRNA expression profile and thus has an impacttumourumor cell processes [15]. Although later studies find that DICER also exists in the nucleus, it is generally believed that the changes in cellular physiological processes caused by DICER are mainly due to its reduction in the cytoplasm, which affects the synthesis of miRNAs [6]. By contrast, in this study we reported that the protein level of DICER in the nucleus was regulated by the ubiquitination mediated by the USP7‐MDM2 axis, resulting in changes in the protein level of DICER in the nucleus, which did not affect the miRNA expression profile but participated in the DNA damage response to regulate tumourumor cell phenotype.

Previous studies have shown that USP7 and MDM2 play a critical role in the regulation of the p53 pathway. MDM2 is the substrate of USP7, and p53 can be ubiquitinated by MDM2 [32]. Similarly, we identified that USP7 downregulated DICER protein by stabilization of MDM2, which acted as a new E3 ligase of DICER. USP7 and MDM2 are both nucleocytoplasmic shuttle proteins. Generally, their expressions are typically higher in the cell nucleus than in the cytoplasm. This is because they perform critical cellular functions in the nucleus, such as protein modifications, gene transcription regulation and DNA damage repair. While DICER is primarily located in the cytoplasm, with a small fraction present in the cell nucleus. There have also been a number of previously published articles finding that DICER in the nucleus is involved in DNA damage repair. Therefore, it is reasonable that the USP7‐MDM2‐Dicer axis works in the nucleus. This finding suggests that the regulatory axis USP7‐MDM2 may have more substrates in cells and play a broader regulatory role, rather than mainly relying on the regulation of p53.

## 5 Conclusions

In summary, our findings demonstrate that in the nucleus, USP7 negatively regulates DICER at the protein level forming a new regulatory axis, which affects DNA damage repair and then regulates tumour growth, and that MDM2 is a new ubiquitin E3 ligase of DICER, which indirectly mediates the negative regulation of DICER by USP7.

## Conflict of interest

The authors declare that they have no known competing financial interests or personal relationships that could have appeared to influence the work reported in this article.

## Author contributions

JY and XL designed the study; XL, RL and QY performed most of the experiments; JFH, CH, YC, ZZ, JYH, LL, RC, YW and JH helped with experiments and provided technical support; JY, XZ and RX analyzed the data; JY and XL wrote the manuscript. All the authors read and approved the final manuscript.

### Peer review

The peer review history for this article is available at https://www.webofscience.com/api/gateway/wos/peer‐review/10.1002/1878‐0261.13543.

## Supporting information


**Fig. S1.** USP7 interacts with DICER and downregulates the DICER protein expression.

**Fig. S2.** Knockdown of USP7 inhibits cancer progression by upregulation of DICER.

**Fig. S3.** USP7‐DICER axis regulates the mRNA expression profile.

**Fig. S4.** USP7‐DICER axis regulates the DNA damage response.

**Fig. S5.** MDM2 ubiquitinates and degrades DICER.

**Table S1.** The list of primer sequences for plasmid constructions, point mutations, shRNAs and qRT–PCR.

**Table S2.** The list of proteins interacted with DICER by the MS analysis.

## Data Availability

The miRNA‐Seq and RNA‐Seq data generated in this study have been deposited in GEO with the following accession number: GSE225307. The MS proteomics data have been deposited to the ProteomeXchange Consortium (http://proteomecentral.proteomexchange.org) via the iProX partner repository with the dataset identifier PXD040295. All data generated or analyzed during the current study are available from the corresponding author upon reasonable request.
