# Supplementary material for: USP7 reduces the level of nuclear DICER, impairing DNA damage response and promoting cancer progression
Source: Mol Oncol. 2023 Nov 2;18(1):170–89. doi: 10.1002/1878-0261.13543 (PMC10766207; doi:10.1002/1878-0261.13543)
Supplement: Supplementary file 1 — Fig. S1. USP7 interacts with DICER and downregulates the DICER protein expression. [file MOL2-18-170-s007.pdf]

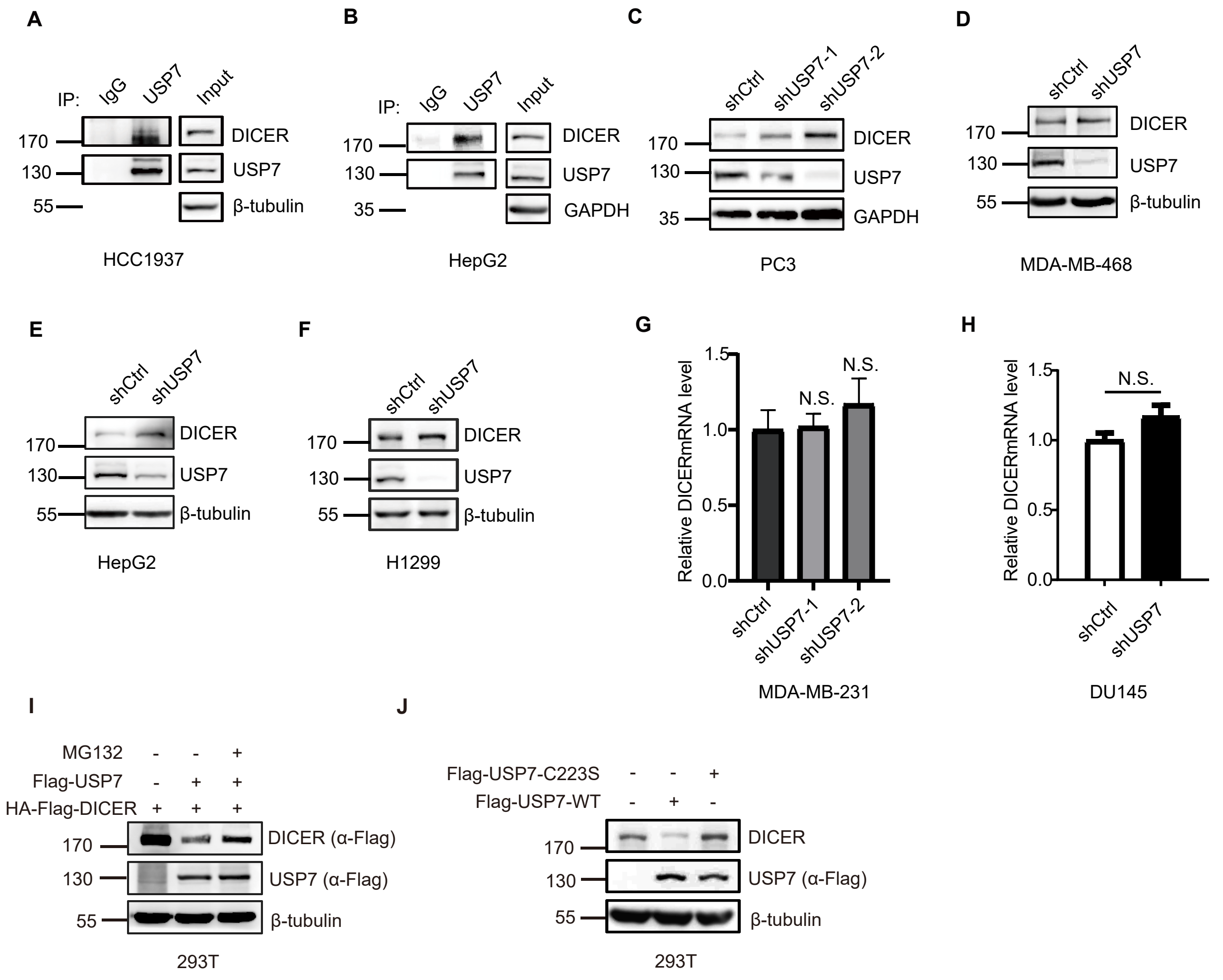

Fig. S1 USP7 interacts with DICER and downregulates DICER protein expression.

A, B Co-IP showing the interaction of endogenous USP7 and DICER in HCC1937 (**A**) and HepG2 (**B**) cells, detected by WB. **C-F** Expression of DICER elevated after USP7 knockdown in PC3 (**C**), MDA-MB-468 (**D**), HepG2 (**E**), and H1299 (**F**) cell lines, detected by WB. **G, H** The mRNA expression of DICER by qPCR in MDA-MB-231 (**G**) and DU145 (**H**) stable cell lines. **I** 293T cells transiently co-transfected Flag-USP7 and HA-Flag-DICER were treated with MG132 (20 μ M) for 6 h, and then followed by WB . **J** Western blotting analysis for 293T cells transfected with Flag-USP7-WT or an inactive mutant Flag-USP7-C223S.
