# Supplementary material for: USP7 reduces the level of nuclear DICER, impairing DNA damage response and promoting cancer progression
Source: Mol Oncol. 2023 Nov 2;18(1):170–89. doi: 10.1002/1878-0261.13543 (PMC10766207; doi:10.1002/1878-0261.13543)
Supplement: Supplementary file 2 — Fig. S2. Knockdown of USP7 inhibits cancer progression by upregulation of DICER. [file MOL2-18-170-s006.pdf]

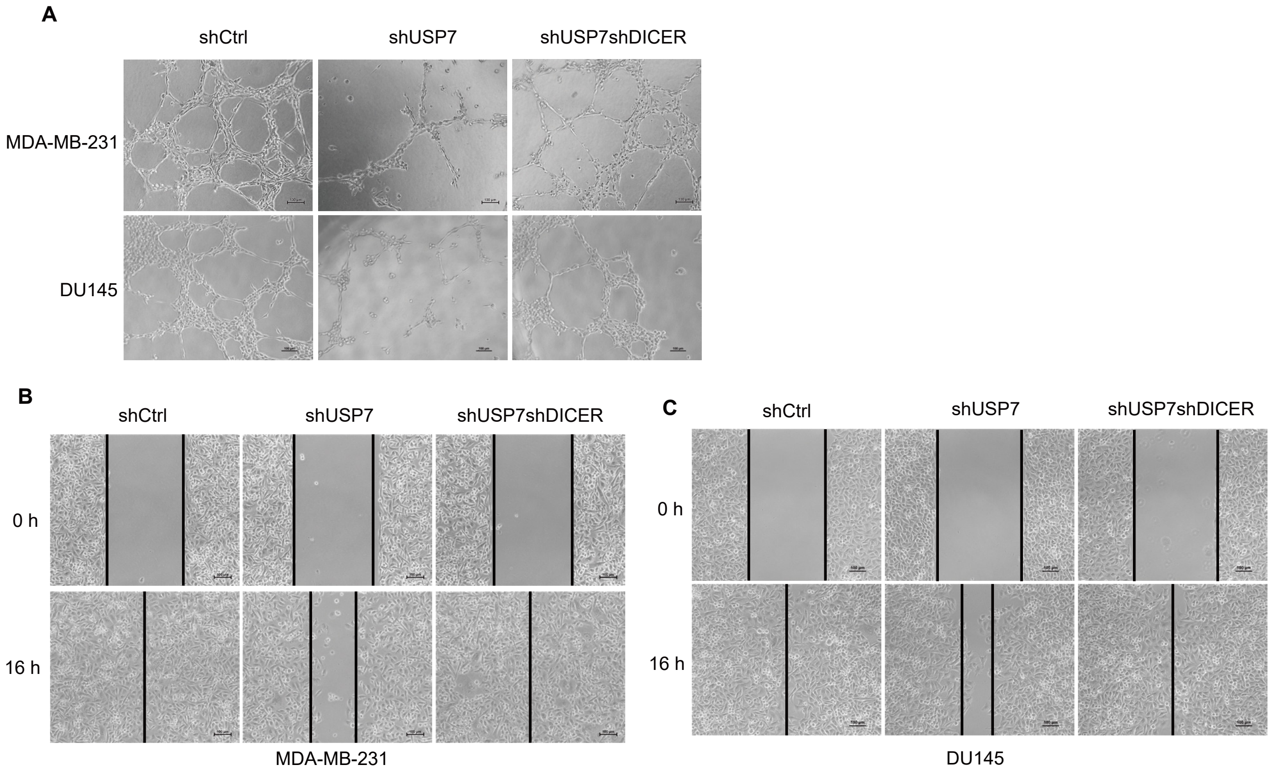

Fig. S2 Knockdown of USP7 inhibits cancer progression by upregulation of DICER.
A Vasculogenic mimicry assay in stable MDA-MB-231 and DU145 cell lines, photographs were taken 12 h later, scale: 100 μm. **B-C** Wound healing assay, photographs were taken at 0 h and 16 h later in MDA-MB-231 (**B**) and DU145 (**C**) cells, scale: 100 μm.
