# Supplementary material for: USP7 reduces the level of nuclear DICER, impairing DNA damage response and promoting cancer progression
Source: Mol Oncol. 2023 Nov 2;18(1):170–89. doi: 10.1002/1878-0261.13543 (PMC10766207; doi:10.1002/1878-0261.13543)
Supplement: Supplementary file 3 — Fig. S3. USP7‐DICER axis regulates the mRNA expression profile. [file MOL2-18-170-s001.pdf]

Liu_Fig.S3

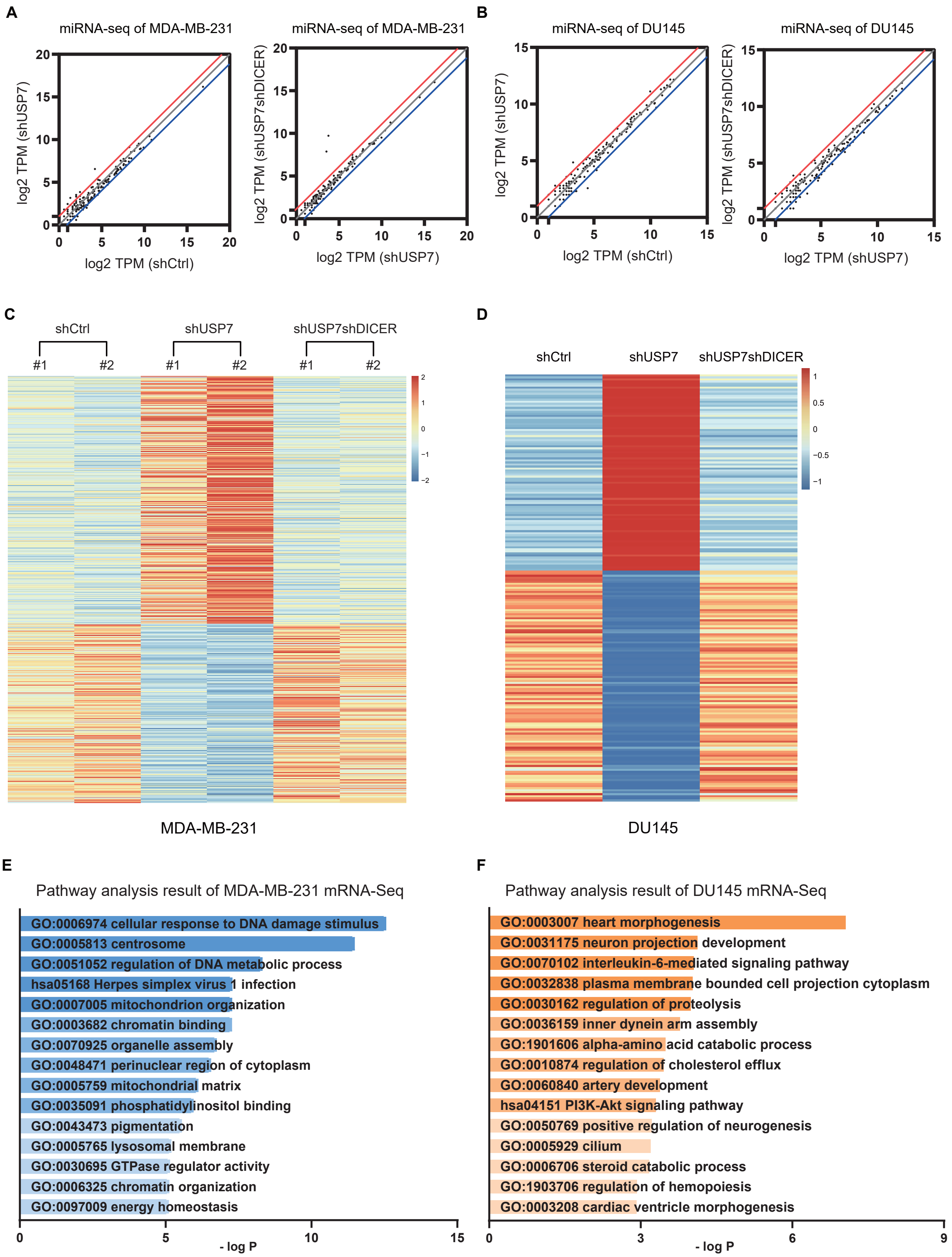
A
miRNA-seq of MDA-MB-231
log2 TPM (shUSP7)
log2 TPM (shCtrl)
miRNA-seq of MDA-MB-231
log2 TPM (shUSP7shDICER)
log2 TPM (shUSP7)
B
miRNA-seq of DU145
log2 TPM (shUSP7)
log2 TPM (shCtrl)
miRNA-seq of DU145
log2 TPM (shUSP7shDICER)
log2 TPM (shUSP7)
C
shCtrl
shUSP7
shUSP7shDICER
#1
#2
#1
#2
#1
#2
MDA-MB-231
D
shCtrl
shUSP7
shUSP7shDICER
DU145
E
Pathway analysis result of MDA-MB-231 mRNA-Seq
GO:0006974 cellular response to DNA damage stimulus
GO:0005813 centrosome
GO:0051052 regulation of DNA metabolic process
hsa05168 Herpes simplex virus 1 infection
GO:0007005 mitochondrion organization
GO:0003682 chromatin binding
GO:0070925 organelle assembly
GO:0048471 perinuclear region of cytoplasm
GO:0005759 mitochondrial matrix
GO:0035091 phosphatidylinositol binding
GO:0043473 pigmentation
GO:0005765 lysosomal membrane
GO:0030695 GTPase regulator activity
GO:0006325 chromatin organization
GO:0097009 energy homeostasis
- log P
F
Pathway analysis result of DU145 mRNA-Seq
GO:0003007 heart morphogenesis
GO:0031175 neuron projection development
GO:0070102 interleukin-6-mediated signaling pathway
GO:0032838 plasma membrane bounded cell projection cytoplasm
GO:0030162 regulation of proteolysis
GO:0036159 inner dynein arm assembly
GO:1901606 alpha-amino acid catabolic process
GO:0010874 regulation of cholesterol efflux
GO:0060840 artery development
hsa04151 PI3K-Akt signaling pathway
GO:0050769 positive regulation of neurogenesis
GO:0005929 cilium
GO:0006706 steroid catabolic process
GO:1903706 regulation of hemopoiesis
GO:0003208 cardiac ventricle morphogenesis
- log P

Fig.S3 USP7-DICER axis regulates mRNA expression profile.

A, B The Scatter plots showing analysis of miRNA-Seq results in MDA-MB-231 (**A**) and DU145 (**B**) stable cell lines, the red lines indicate the range of fold change > 2, the blue lines indicate the range of fold change < -2 (shUSP7 over shCtrl, or shUSP7shDICER over shUSP7), TPM > 1. **C, D** Heatmaps showing mRNAs regulated by USP7-DICER axis in MDA-MB-231 (2 samples per group) (**C**) and DU145 (1 sample per group) (**D**) stable cell lines, FPKM > 1, fold change > 1.5. **E, F** GO and KEGG pathway enrichment analysis of genes regulated by USP7-DICER axis in MDA-MB-231 (**E**) and DU145 (**F**) stable cell lines, from mRNA-Seq results, FPKM > 1, Fold change > 1.5.
