# Supplementary material for: USP7 reduces the level of nuclear DICER, impairing DNA damage response and promoting cancer progression
Source: Mol Oncol. 2023 Nov 2;18(1):170–89. doi: 10.1002/1878-0261.13543 (PMC10766207; doi:10.1002/1878-0261.13543)
Supplement: Supplementary file 4 — Fig. S4. USP7‐DICER axis regulates the DNA damage response. [file MOL2-18-170-s005.pdf]

Liu_Fig.S4

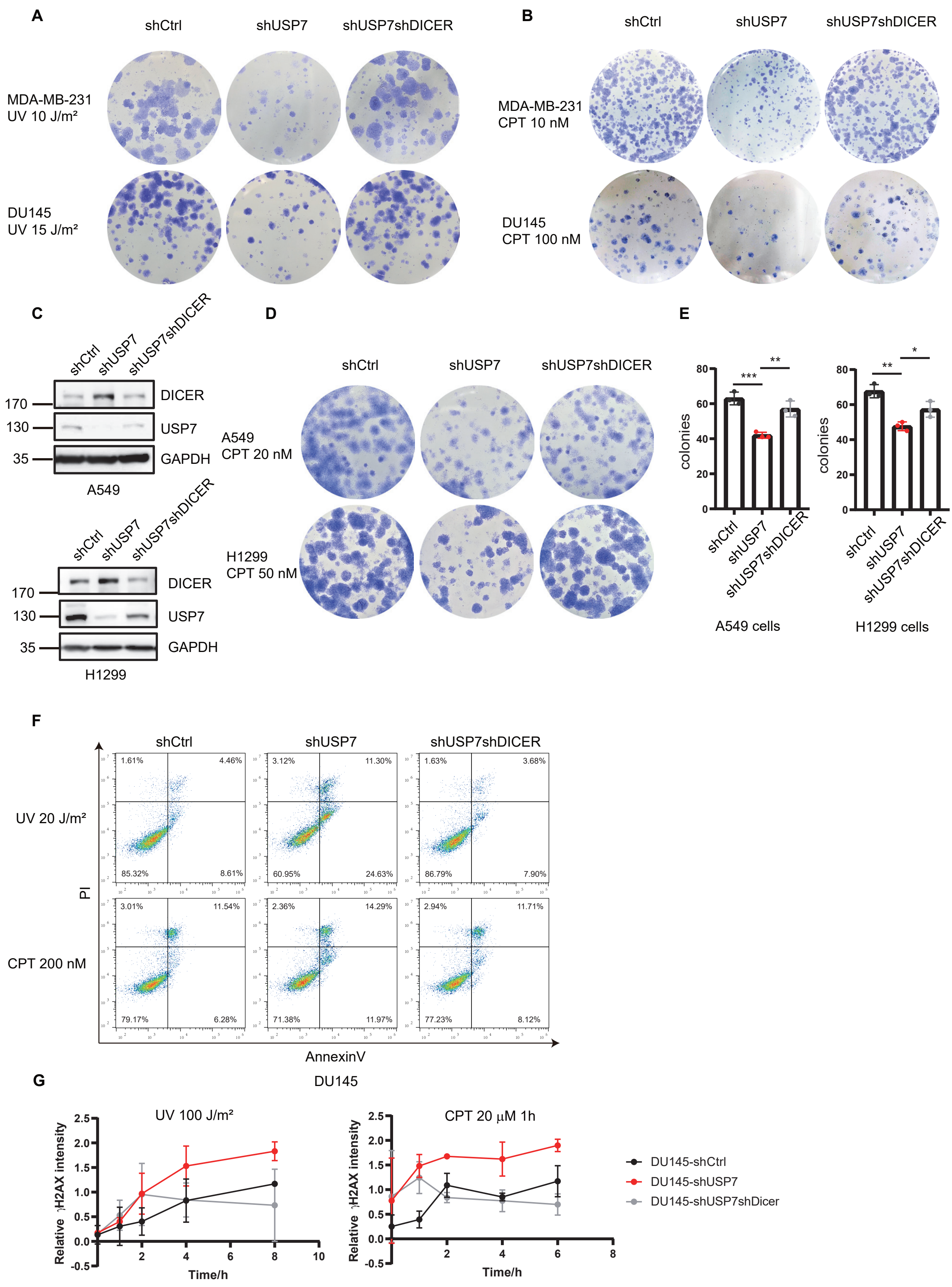

A
shCtrl
shUSP7
shUSP7shDICER
MDA-MB-231
UV 10 J/m²
DU145
UV 15 J/m²
B
shCtrl
shUSP7
shUSP7shDICER
MDA-MB-231
CPT 10 nM
DU145
CPT 100 nM
C
shCtrl
shUSP7
shUSP7shDICER
170
130
35
DICER
USP7
GAPDH
A549
H1299
D
shCtrl
shUSP7
shUSP7shDICER
A549
CPT 20 nM
H1299
CPT 50 nM
E
colonies
A549 cells
H1299 cells
F
shCtrl
shUSP7
shUSP7shDICER
UV 20 J/m²
CPT 200 nM
PI
AnnexinV
1.61%
4.46%
85.32%
8.61%
3.12%
11.30%
60.95%
24.63%
1.63%
3.68%
86.79%
7.90%
3.01%
11.54%
79.17%
6.28%
2.36%
14.29%
71.38%
11.97%
2.94%
11.71%
77.23%
8.12%
G
DU145
UV 100 J/m²
CPT 20 μM 1h
Relative γH2AX intensity
Time/h
DU145-shCtrl
DU145-shUSP7
DU145-shUSP7shDicer

H

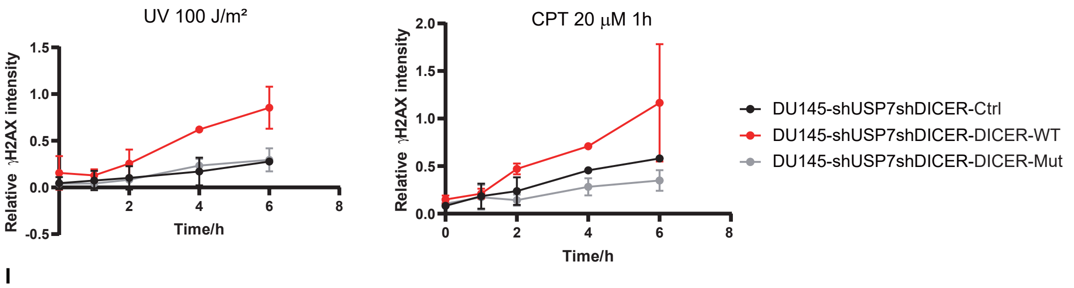

I

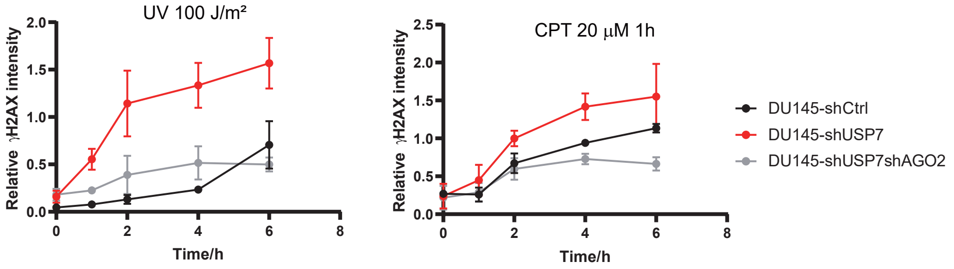

Fig.S4 USP7-DICER axis regulates DNA damage response.

A, B Clonal survival of MDA-MB-231 and DU145 stable cell lines under defined intensity of UV (**A**) or specified concerntration of CPT (**B**) treatments. **C** Construction of shUSP7shDICER stable cell lines using A549 and H1299 cells. **D** Clonal survival of A549 and H1299 stable cell lines under specified concerntration of CPT treatment. **E** Statistics of the colonies in figure (**D**), n=3, *p < 0.05, **p < 0.01, ***p < 0.001 . **F** Detection of apoptosis levels of DU145 stable cell lines under defined intensity of UV or concerntration of CPT treatments, analyzed by flow cytometry. **G-I** Quantification results of γH2AX relative intensity in Fig.4F, Fig.4H, and Fig.4I, respectively, n=3.
