# Supplementary material for: USP7 reduces the level of nuclear DICER, impairing DNA damage response and promoting cancer progression
Source: Mol Oncol. 2023 Nov 2;18(1):170–89. doi: 10.1002/1878-0261.13543 (PMC10766207; doi:10.1002/1878-0261.13543)
Supplement: Supplementary file 5 — Fig. S5. MDM2 ubiquitinates and degrades DICER. [file MOL2-18-170-s004.pdf]

Liu_Fig.S5

A

E3 ligase substrates	Published year
MDM2	2004
UHRF1	2015
ICP0	1997
RAD18	2016
PCNA	2015
RNF168	2015
RNF169	2017
RING1B	2010
MEL18	2010
BMI1	2010
MARCH7	2008
NEDD4L	2020

B

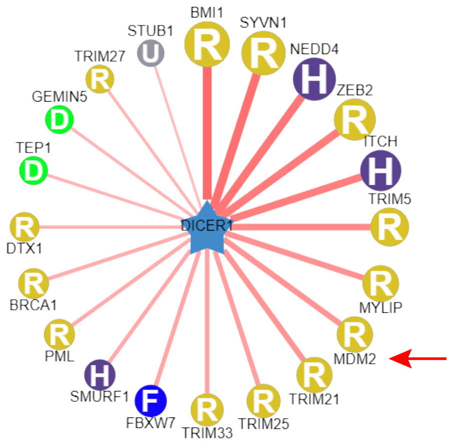

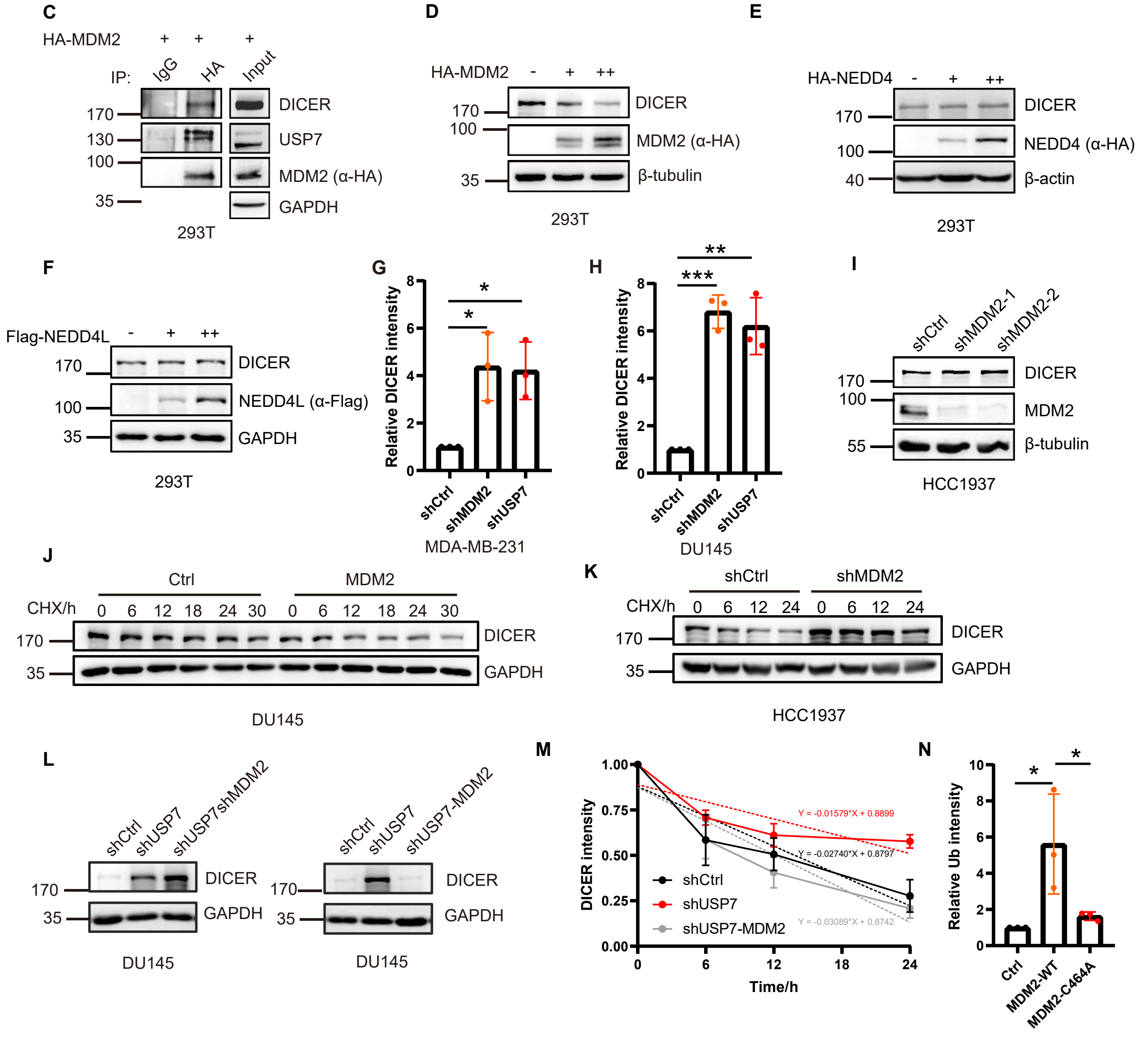

O

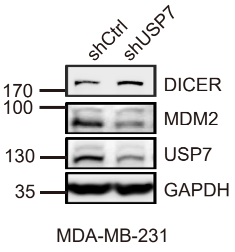

Fig.S5 MDM2 ubiquitinates and degrades DICER.

A E3 ligases which are substrates of deubiquitinase USP7 reported in the literature. **B** Potential E3 ligases of DICER predicted by UbiBrowser. **C** The interaction of overexpressed HA-MDM2 with DICER was detected in 293T cells by Co-IP/WB. **D** Transiently expressed MDM2 in 293T cells, resulted in the reduction of endogenous DICER. **E,F** Endogenous DICER protein expression level observed in 293T cells with gradiently overexpressed HA-NEDD4 (**E**) or Flag-NEDD4L (**F**) plasmids, detected by WB. **G,H** The quantification result of Fig.6E,F, n=3. **I** DICER protein expression level observed in HCC1937-shMDM2 cells, detected by WB. **J** After 0, 6, 12, 18, 24,30 hours of CHX (100 μg/mL) treatment, endogenous DICER protein expression level was observed in DU145-MDM2 and control cell lines, detected by WB. **K** After 0, 6, 12, 24 hours of CHX (100 μg/mL) treatment, endogenous DICER protein expression level was observed in HCC1937-shMDM2 and control cell lines, detected by WB. **L** The knockdown (left) or Over-expression MDM2 (right) stable cell lines at shUSP7 conditions. **M** Quantification result of DICER intensity in Fig.6G, n=3. **N** The quantification result of Ub intensity in Fig.6H, n=3. **O** Western blotting analysis of the protein levels of MDM2 and DICER in USP7-knockdown MDA-MB-231 cells.
