# Supplementary material for: USP7 reduces the level of nuclear DICER, impairing DNA damage response and promoting cancer progression
Source: Mol Oncol. 2023 Nov 2;18(1):170–89. doi: 10.1002/1878-0261.13543 (PMC10766207; doi:10.1002/1878-0261.13543)
Supplement: Supplementary file 6 — Table S1. The list of primer sequences for plasmid constructions, point mutations, shRNAs and qRT–PCR. [file MOL2-18-170-s003.pdf]

Table S1. The list of primer sequences for plasmid constructions, point mutations, shRNAs, and qRT-PCR

Primers	Sequence(5'-3')
shUSP7-1-F	CCGGTGTATCTATTGACTGCCCTTTCTCGAGAAAGGGCAGTCAATAGATACATTTTTG
shUSP7-1-R	AATTCAAAAATGTATCTATTGACTGCCCTTTCTCGAGAAAGGGCAGTCAATAGATACA
shUSP7-2-F	CCGGCCTGGATTTGTGGTTACGTTACTCGAGTAACGTAACCACAAATCCAGGTTTTTG
shUSP7-2-R	AATTCAAAAACCTGGATTTGTGGTTACGTTACTCGAGTAACGTAACCACAAATCCAGG
USP7-C223S-F	AATCAGGGAGCGACTTCTTACATGAAC
USP7-C223S-R	CTTTAAGCCGACGTAGCCTGTGTG
shDicer-F	CCGGGCTCGAAATCTTACGCAAATACTCGAGTATTTGCGTAAGATTTCGAGCTTTTTG
shDicer-R	AATTCAAAAAGCTCGAAATCTTACGCAAATACTCGAGTATTTGCGTAAGATTTCGAGC
MDM2-Flag-F	TTTTGACCTCCATAGAAGATTCTAGAATGGATTACAAGGATGACGACGATAATGCAATACCAACATGTCT
MDM2-R	CGGAGCGATCGCAGATCCTTGCGGCCGCCTAGGGGAAATAAGTTAGCAC
shMDM2-1-F	CCGGCTCAGCCATCAACTTCTAGTACTCGAGTACTAGAAGTTGATGGCTGAGTTTTTG
shMDM2-1-R	AATTCAAAAACTCAGCCATCAACTTCTAGTACTCGAGTACTAGAAGTTGATGGCTGAG
shMDM2-2-F	CCGGATTATCTGGTGAACGACAAAGCTCGAGCTTTGTCGTTCACCAGATAATTTTTTG
shMDM2-2-R	AATTCAAAAAATTATCTGGTGAACGACAAAGCTCGAGCTTTGTCGTTCACCAGATAAT
MDM2-C464A-F	ATGGCCTGCTTTACAGCTGCAAAGA
MDM2-C464A-R	AAGATGTCCTGTTTTGCCATG
MDM2-NLS-mut-F	TCTGGTGAACGACAAACAAAACTCCAC
MDM2-NLS-mut-R	TAATTCATCTGAATTTTCTTC
MDM2-NES-mut-F	GATGAAAGCCTGGCTGCATGTGTAGCAAGGGAGATATG
MDM2-NES-mut-R	AAAGGAAAGGGAAATACTATC
shAGO2-F	CCGGTCTATGAACTCAGGGCTTTAAACTCGAGTT TAAAGCCCTGAGTTCATAGTTTTTTG
shAGO2-R	AATTCAAAAAACTATGAACTCAGGGCTTTAAACT CGAGTTTAAAGCCCTGAGTTCATAGA
Q-DICER-F	CTGGCAGGTGTACTATCCCA
Q-DICER-R	CCTGTAACTTCGACCAACACC
